# Long term effects on human plasma lipoproteins of a formulation enriched in butter milk polar lipid

**DOI:** 10.1186/1476-511X-8-44

**Published:** 2009-10-16

**Authors:** Lena Ohlsson, Hans Burling, Åke Nilsson

**Affiliations:** 1 Department of Clinical Sciences, Medicine (Gastroenterology and Nutrition), Lund University Hospital, S221 85 Lund, Sweden; 2 Arla Foods AB, Scheelegatan, Lund, Sweden

## Abstract

**Background:**

Sphingolipids (SL), in particular sphingomyelin (SM) are important components of milk fat polar lipids. Dietary SM inhibits cholesterol absorption in rats (Nyberg et al. *J Nutr Biochem*. 2000) and SLs decrease both cholesterol and TG concentrations in lipid- and cholesterol fed APOE*3Leiden mice (Duivenvoorden et al. *Am J Clin Nutr*. 2006). This human study examines effects of a butter milk formulation enriched in milk fat globule membrane material, and thereby in SLs, on blood lipids in healthy volunteers. In a four week parallel group study with 33 men and 15 women we examined the effects of an SL-enriched butter milk formulation (A) and an equivalent control formulation (B) on plasma lipid levels. Plasma concentrations of HDL and LDL cholesterol, triacylglycerols (TG), apolipoproteins AI and B, and lipoprotein (a) were measured. The daily dose of SL in A was 975 mg of which 700 mg was SM. The participants registered food and drink intake four days before introducing the test formula and the last four days of the test period.

**Results:**

A daily increase of SL intake did not significantly influence fasting plasma lipids or lipoproteins. In group B TG, cholesterol, LDL, HDL and apolipoprotein B concentrations increased, however, but not in group A after four weeks. The difference in LDL cholesterol was seen primarily in women and difference in TG primarily in men. No significant side effects were observed.

**Conclusion:**

The study did not show any significant decrease on plasma lipids or lipoprotein levels of an SL-enriched formulation containing 2-3 times more SL than the normal dietary intake on cholesterol, other plasma lipids or on energy intake. The formulation A may, however, have counteracted the trend towards increased blood lipid concentrations caused by increased energy intake that was seen with the B formulation.

## Background

High concentrations of plasma cholesterol, especially low density lipoproteins (LDL) and/or triglycerides (TG) and lipoprotein a (Lp (a)) [1] are well known risk factors for cardiovascular disease (CVD) whereas HDL levels are inversely correlated to risk. Plasma lipid and lipoprotein levels are determined by genetic predisposition as well as lifestyle and dietary levels can be influenced by numerous dietary factors acting via several mechanisms. To reduce intestinal cholesterol absorption by dietary factors is an important way to improve blood lipid concentrations. A successful example is that foods containing plant sterols can reduce LDL cholesterol levels by 10-20% [2].

Sphingomyelin (SM) is a polar lipid in the milk fat globule membrane (MFGM) and accounts for 25% of the membrane lipids in bovine milk [3]. SM is also a precursor to the bioactive sphingolipid signaling molecules ceramide, sphingosine and sphingosine-1-phospate [4]. Both dietary and membrane SM interact strongly with cholesterol [5]. Studies on rats have shown that dietary SM inhibits cholesterol absorption and the inhibition is more effective with milk SM than egg SM [6,7]. Dietary SL decreased both cholesterol and TG concentrations in lipid- and cholesterol fed ApoE Leyden mice [8].

Milk fat contains 2-3% of polar lipids and the Western dietary intake of SM from bovine milk is approximately 4-12 mg/dl [9]. Buttermilk and whey are two liquid remains from manufacturing of butter and cheese, which have in recent years gained more interest as a source of bioactive compounds such as MFGM [10]. During the process of churning and cheese making an excess of polar MFGM membrane material is released in the buttermilk and whey. New technologies for enrichment of MFGM, including the one developed by Arla Foods and used in this study, have emerged. There are no previous studies on long term intake of SL in humans due to lack of suitable formulations.

In this study we examine the effects of a tasty milk drink-like formulation enriched in polar lipids from buttermilk. The effects on plasma cholesterol, TG, HDL, LDL and apolipoproteins A1 (apoA1), B (apoB) and Lp(a) over 4 weeks daily intake of 2-3 times the normal daily intake of SL were examined. The main hypothesis was that the long term intake of SM enriched drink would inhibit intestinal cholesterol uptake with lowered total plasma- and LDL-cholesterol as outcome. We also asked whether TG metabolism is beneficially influenced, as in the apoE Leyden mice [8].

## Methods

### Drinks

The test drink A used in this study were based on processed and enriched butter milk and contains 4,0% Lacprodan PL-20 powder from Arla Foods amba containing 20% milk phospholipids originating from the serum phase of butter oil production (Table 1). The test drink (A) contains 700 mg SM, 180 mg glucoceramides, and 95 mg gangliosides (mainly GD3). Total amount of SL 975 mg in one portion of about 350 ml. A placebo drink (B) was made by 5,82% skim milk powder containing 37% protein in which the phospholipids have been replaced by 2/3 of egg PL isolate by Fresenius GmbH and 1/3 butter oil. The placebo drink contains 119 mg of SL per portion. The cholesterol content was adjusted to be the same in both drinks, i.e. 11 mg per 100 ml or 38 mg per serving.

**Table 1:** Composition of test drink A and placebo drink B.

	**A (per 100 g)**	**A (daily )**	**B (per 100 g)**	**B (daily)**
Total lipid of which polar lipids SL	1,12 g	3,7 g	1,12 g	3,7 g
	800 mg	2,8 g	768 mg	2,7 g
	295 mg	975 mg	34 mg	119 mg

Protein	2,4 g	8,4 g	2,2 g	7,7 g

Sucrose	4,2 g	14,7 g	4,2 g	14,7 g

Lactose	2,7 g	9,5 g	2,7 g	9,5 g

Maltodextrin	2,0 g	7 g	2,0 g	7 g

Cholesterol	11 mg	38 mg	11 mg	38 mg

Total Energy	230 KJ	806 KJ	230 KJ	806 KJ

The amounts are shown per 100 g and the daily intake of 350 ml. The polar lipids in A are milk phospholipids and in B 66% are egg phospholipids.

The drinks has been subjected to heat treatment at 143C for 6 s and poured into Tetra Brick containers under aseptic conditions. The sterility of the drink has been verified by certified control laboratory. Test and placebo drink was manufactured at Trenums Industries, Tingsryd, Sweden, under supervision of Arla-experts. Trenums is an approved facility for manufacturing of food items. The drinks were stored in refrigerator and it is recommended to shake it a little before consumption.

### Study design

This study includes 48 healthy persons between 20 and 65 years of age, that were healthy according to their knowledge, and as indicated by the initial laboratory screening described below (Table 2). The participants were distributed as follows: 33 men of whom 20 had test drink A, and 15 women of whom 9 had test drink A.

**Table 2:** Characteristics of the 48 subjects.

	**Men n = 33**		**Women n = 15**	
	
	**A(20)**	**B(13)**	**A(9)**	**B(6)**
Age	47,4 ± 2,9	42,9 ± 4,6	45,1 ± 3,6	43,3 ± 5,7

BMI	24,1 ± 0,4	24,9 ± 0,5	24,0 ± 1,0	24,5 ± 1,6

Length, m	1,78 ± 1,6	1,79 ± 1,6	1,69 ± 1,9	1,68 ± 3,5

Weight, kg	76,8 ± 1,9	80,2 ± 2,4	68,3 ± 3,1	69,8 ± 6,0

Starting TG in mmol/L	1,15 ± 0,15	0,96 ± 0,12	0,92 ± 0,13	0,67 ± 0,07

Starting CHOL in mmol/L	4,86 ± 0,20	4,49 ± 0,23	4,70 ± 0,29	4,47 ± 0,23

Starting LDL in mmol/L	3,31 ± 0,18	2,95 ± 0,22	3,09 ± 0,27	2,68 ± 0,15

Starting HDL in mmol/L	1,33 ± 0,07	1,34 ± 0,08	1,50 ± 0,09	1,80 ± 0,20

Starting ApoA1 in g/L	1,39 ± 0,03	1,37 ± 0,05	1,51 ± 0,07	1,60 ± 0,14

Starting ApoB in g/L	0,88 ± 0,05	0,79 ± 0,05	0,82 ± 0,06	0,69 ± 0,03

Values are mean ± SEM (number in each group).

Voluntary participants were recruited through announcements on local intranet and advertisement in different work places in Lund. All participants gave their written informed consent prior to the study. The study was approved by the Regional Human Ethics Committee of Lund-Malmö.

The study was over a five week period of which the first week was a run in period when they kept a compulsory food diary of their intake for 4 days. The diary was designed by the Department of Clinical Nutrition at Lund University Hospital. After the first week participants drank the test drink or placebo drink every day for four weeks. The participants got a supply of 28 portions to be kept refrigerated at home and consume half a portion (about 175 ml) in the morning and the last half in the evening. Both test drink and placebo drink were well tolerated and palatable.

The participants begin the five week study by providing fasting blood samples. The morning the same day they start with test/placebo drink they also provide blood samples. Blood samples are taken again after two weeks intake of test/placebo drink and again on the last day of the study. The participants give blood samples all together four times during the study and all of them are taken fasting in the morning at the Department of Clinical Chemistry, University Hospital of Lund.

Throughout the study the participants continued their normal life with no restrictions. They were instructed to report intake of alcohol and pharmaceutical drugs but they were not instructed to report exercise.

### Analysis

Fasting blood samples were analyzed for TG, total cholesterol, HDL, LDL, apoA1 and apoB as well as for liver values, CRP, hemoglobin, white blood cell count and platelet count at Department of Clinical Chemistry, University Hospital of Lund, Sweden . Two ml EDTA plasma was kept frozen for further analysis of Lp (a).

All participants kept the diaries according to instructions and the intake was analyzed by the use of Dietist XP software with the Swedish National Food Administration database (20080306) (Kost och Näringsdata, Sweden). Dietary intakes were related to recommended Swedish dietary guidelines [11].

### Statistics

Values from clinical data are presented in figures as mean ± SEM of normalized values, however all statistical analyses are done with absolute values. Results of the statistical evaluation are presented in the text. Statistical analysis of the three different time points within each group were done with repeated measures ANOVA and Bonferroni as post test. Comparisons of data before and after intake within each group was done with paired students t-test and unpaired t-test was used to calculate if there were any difference between lipid concentrations after the study period for group A vs. group B. Values are considered different for * p < 0,05, ** p < 0,001, *** p < 0,0001. Results from dietary intake are mean of four days intake from each person. Differences in intake both with and without test drink are analyzed with paired Students t-test. Graph Pad Prism 5. GraphPad Software Inc. San Diego, USA was used as soft ware.

## Results

### Plasma lipids

**Figure 1:**
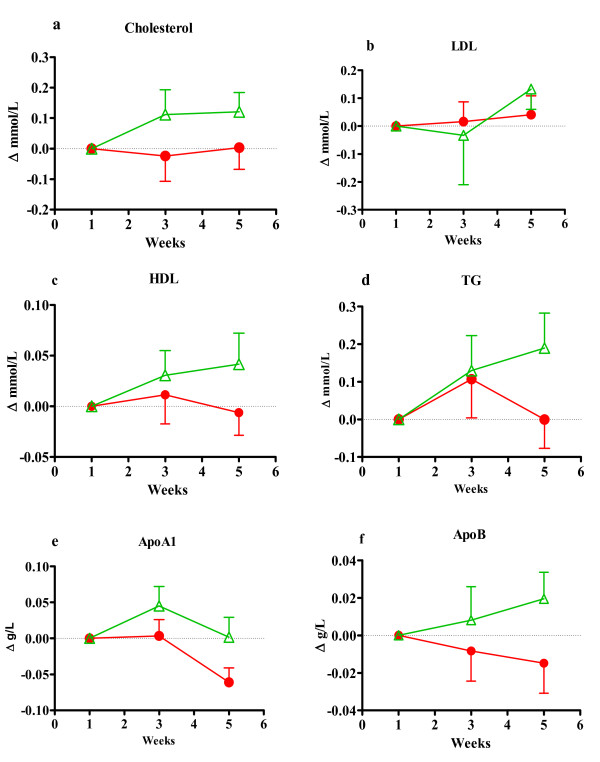
**This figure shows plasma lipids in all participants as differences from starting value**. Figure 1a) Shows total plasma cholesterol, b) Plasma LDL cholesterol, c) plasma HDL cholesterol, d) plasma triacylglycerol, e) apolipoprotein A1 and f) shows apolipoprotein B. A-d are in mmol/L and e-f in g/L. All values are mean ± SEM at fasting state. Red label represents the group taking test drink A, n = 29 and green labels represent the group taking placebo drink B, n = 19.

**Figure 2:**
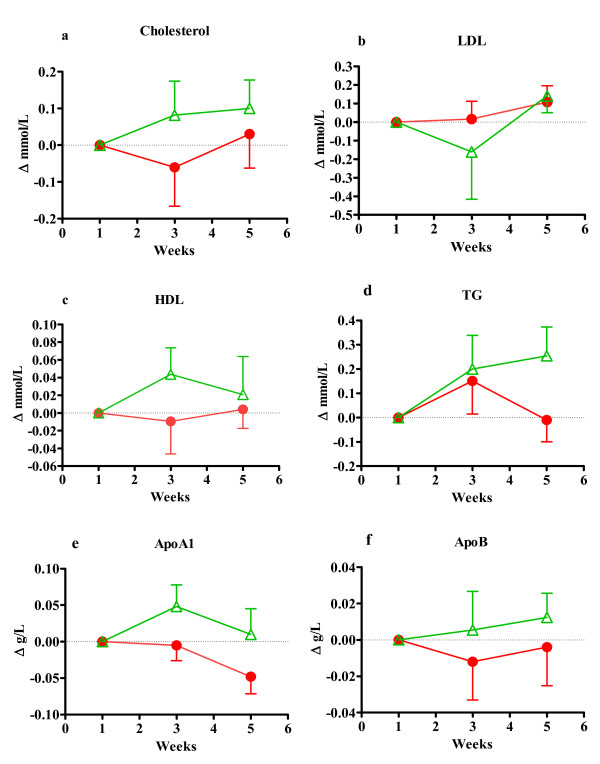
**This figure shows plasma lipids in men as differences from starting value**. Figure 2a) Shows total plasma cholesterol, b) Plasma LDL cholesterol, c) plasma HDL cholesterol, d) plasma triacylglycerol, e) apolipoprotein A1 and f) shows apolipoprotein B. A-d are in mmol/L and e-f in g/L. All values are mean ± SEM at fasting state. Red label represents the group taking test drink A, n = 20 and green labels represent the group taking placebo drink B, n = 13.

**Figure 3:**
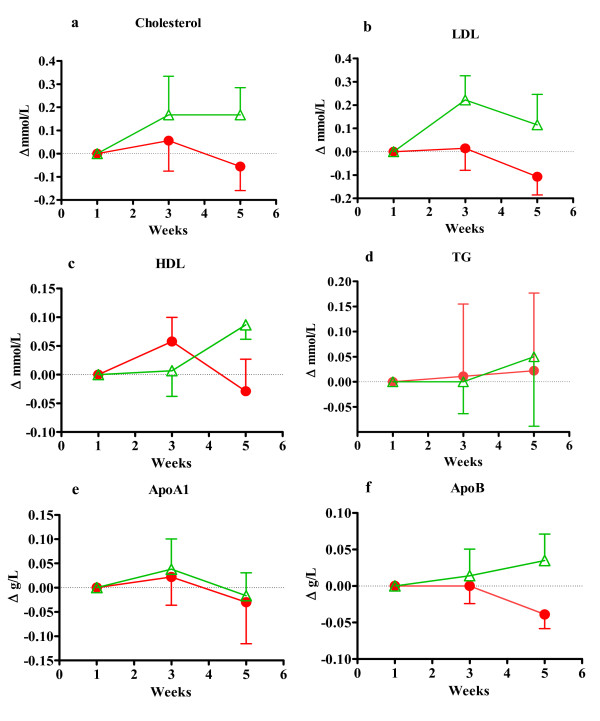
**This figure shows plasma lipids in women as differences from starting value**. Figure 3a) Shows total plasma cholesterol, b) Plasma LDL cholesterol, c) plasma HDL cholesterol, d) plasma triacylglycerol, e) apolipoprotein A1 and f) shows apolipoprotein B. Values in a-d are in mmol/L and e-f in g/L. All values are mean ± SEM at fasting state. Red label represents the group taking test drink A, n = 9 and green labels represent the group taking placebo drink B, n = 6.

**Figure 4:**
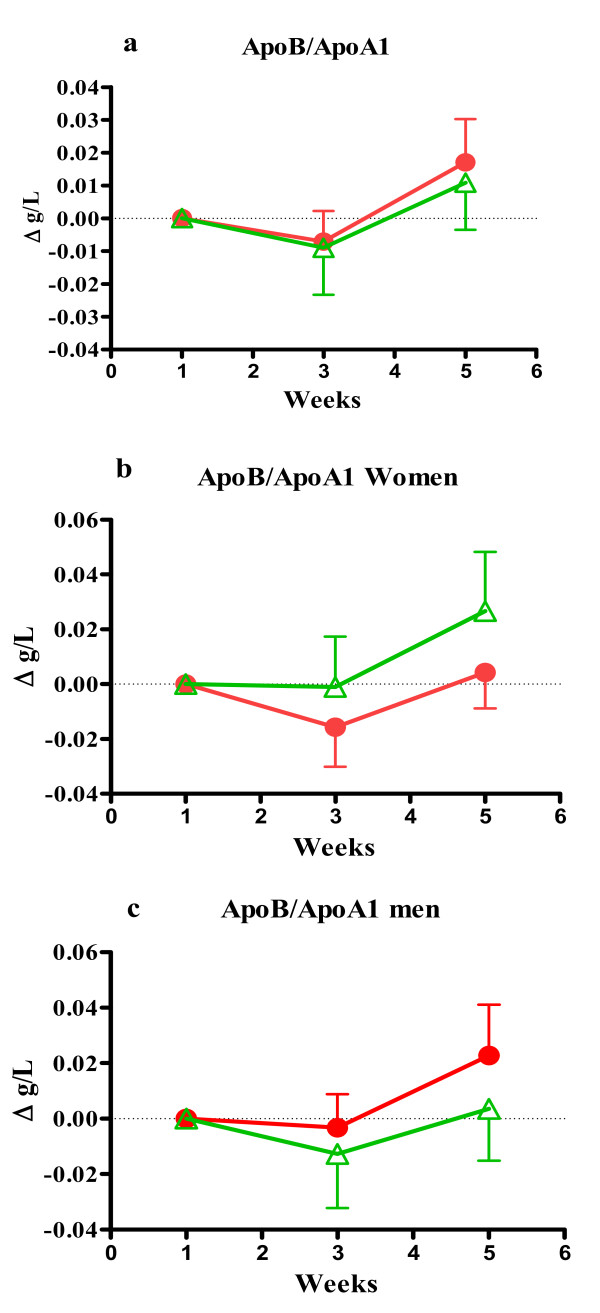
**The figure shows ratios of ApoA1/Apo B**. Figure 4a) shows all participants, b) men, c) women. Values are mean ± SEM and presented as differences from starting value. Red label represent test group A and green label represent placebo group B.

The characteristics of the participants are described in table 2 and did not differ between test- and placebo groups. After four weeks of daily intake of a SM-enriched milk drink (A) or an equivalent SL free control formulation (B) we found no significant changes between group A and B neither in the concentrations of cholesterol, LDL, HDL, TG and apoA1 and apoB nor in the lipid intake (Figures 1, 2, 3 and 4). In all participants (Figure 1) the blood lipid concentrations in group B after intake where generally higher than starting values for total cholesterol (p = 0,072), LDL cholesterol (p = 0,084) and TG (p = 0,098) with much less changes in group A. There was a significant decrease in Apo A1 (p = 0,005) after intake in group A compared to starting value.

In men (Figure 2) there were trends such as increases in plasma cholesterol values and the concentrations of plasma TGs increased by 26% in the placebo group but remained unchanged in the test group but the difference was not significant (p = 0,082) which may be related increased energy intake (Table 3). The cholesterol, HDL LDL concentrations were all slightly elevated by 0,6-4,8% in both placebo and test group. ApoA1 was decreased in group A compared to starting values (p = 0,055) and remained unchanged in group B. Apo B was also slightly increased in group B but not in group A after intake.

**Table 3:** Differences in daily intake of total energy, cholesterol and saturated fat in men four days before and the last four days of the study period.

	**Group A before** **n = 18**	**Group A** **after** **n = 18**	**Difference (%)**	**Group B before** **n = 13**	**Group B after** **n = 13**	**Difference (%)**
Total energy intake (KJ)	9143 ± 474	9040 ± 482	- 1,2	8754 ± 516	9244 ± 469	+ 5,6
Including testdrink		9801 ± 482	+7,2		10005 ± 469*	+14,3

Cholesterol intake (mg)	304 ± 27	286 ± 26	-5,9	323 ± 41	295 ± 41	-8,7
Including test drink		319 ± 26	+4,9		328 ± 41	+1,5

Saturated fat intake (g)	34,3 ± 2,9	33,6 ± 3,5	-2,0	35,2 ± 3,6	35,4 ± 2,82	+ 0,1

Total fat intake (g)	85,4 ± 6,2	83,7 ± 7,6	-1,96	87,7 ± 7,4	86,2 ± 4,2	-1,71
Including testdrink		87,9 ± 7,6	+2,93		90,4 ± 4,2	+2,7

Differences before and after intake in each group was statistically analyzed using students paired t-test. * p < 0,01. ** p = < 0,001. *** p = < 0,0001. The values are mean ± SEM and the values for each subject are mean of four days of food registration.

The difference between group A and B in women (Figure 3) was not significant for any plasma lipid but there were trends such as increases in plasma LDL (p = 0,056) and HDL cholesterol (p = 0,070) in group B compared to group A. There were some differences in initial blood lipid concentrations between group A and B in women. In group B LDL and TG are 13% and 27% lower than in group A but HDL is 20% higher in B than in group A (Table 2) but all concentrations are within normal range both before and after the study. In group B all blood lipid concentrations except apoA1 were increased after the test period and the increase in HDL by 4,8% was statistically different (p = 0,018) compared to starting values. In group A there were decreased concentrations of total cholesterol, LDL and HDL by 1,2, 3,5 and 1,9% respectively which can be related to a decrease of cholesterol intake by 6% but not to increased intake of saturated fat (Table 4). Apolipoprotein B concentration decreased in group A compared to starting concentrations but not significantly (p = 0,083). The increase in plasma TG concentration in both groups coincides with the increased energy and fat intake.

**Table 4:** Differences in daily intake of total energy, cholesterol, saturated- and total fat in women four days before and the last four days of the study period.

	**Group A before** **n = 9**	**Group A after** **n = 9**	**Difference (%)**	**Group B before** **n = 6**	**Group B after** **n = 6**	**Difference (%)**
Total energy intake (KJ)	7448 ± 601	8127 ± 509	+ 9,1	7957 ± 484	8149 ± 898	+ 2,4
Including testdrink		8888 ± 509*	+19,3		8910 ± 898	+12,0

Cholesterol intake (mg)	266 ± 18	250 ± 34	-6,0	256 ± 26	279 ± 65	+ 9,0
Including testdrink		283 ± 34	+6,4		312 ± 65	+21,9

Saturated fat intake (g)	31,4 ± 3,7	34,4 ± 3,2	+9,6	31,0 ± 4,04	35,2 ± 5,9	+ 13,5

Total fat intake (g)	71,7 ± 7,8	82,0 ± 8,1	+14,4	73,2 ± 8,7	78,8 ± 11,7	+7,7
Including testdrink		86,2 ± 8,1	+20,2		83 ± 11,7	+9,8

Differences before and after intake in each group was statistically analyzed using students paired t-test. * p < 0,01. ** p = < 0,001. *** p = < 0,0001. The values are mean ± SEM and the values for each subject are mean of four days of food registration.

There was a minor increase in the apoB/apoA1 ratios in both groups (Figure 4). In men this increase was predominantly in group A as opposed to women where group B had the highest increase after four weeks. The concentrations of Lp (a) varied considerably between below 60 and 489 mg/ml in both men and women but no significant changes were seen after four weeks (Table 5). For women in group A Lp(a) concentration was reduced by 10% but increased in all other groups after four weeks.

**Table 5:** Concentration of plasma lipoprotein (a).

	**Test group before**	**Test group after**	**Difference (%)**	**Placebo group before**	**Placebo group after**	**Difference (%)**
Men	166 ± 51 (16^1^)	182 ± 47	+ 9,6	166 ± 38 (11)	168 ± 70	+ 1,2
Women	127 ± 26 (8^1^)	114 ± 28	- 10,2	165 ± 44 (6)	207 ± 64	+ 25
All	153 ± 35 (24^1^)	159 ± 33	+ 3,9	169 ± 28 (17)	181 ± 34	+ 7,1

Missing values are due to sample hemolysis and could not be measured.

The table shows lipoprotein (a) concentrations in men and women before and after intervention period. The values are mg/L and mean ± SEM (number of subjects).

### Food intake

From analysis of the food diaries before and in the end of the 4 week period the energy intake, cholesterol and fat intake were increased at the end of the test period in both groups, which partly can be explained by the addition of test drink to their normal diet (Table 6). The participants were not instructed to inform us whether the test drink replaced something in their normal diet or if it was just added. The energy intake by men (Table 3) was increased by 5.6% in the placebo group and decreased by 1.2% in the test group. When we include the energy from the test drink the increased intake in group B was significantly higher after four weeks (p = 0.03). In women (Table 4) the energy intake was increased in both groups even without including the test drink. When we include the test drink the intake in group A was significantly higher after four weeks (p = 0.017) (Table 4). The cholesterol intake was reduced by 6% in the test groups of both men and women and by 9% within the men in the placebo group (Table 3 and 4). The women in the placebo group had an increased intake of cholesterol by 9%. When we include the 38 mg cholesterol from the test drinks, all groups increase their cholesterol intake. The fat intake was increased or almost unchanged among all participants in both groups, whether the test drink was included or not (Table 6). The intake of saturated fat was increased among women in both groups but remained stable in men with a minor decrease of 2% in the test group (Table 3 and 4). Overall, the difference in total energy intake, cholesterol and saturated fat intake corresponds to the changes in the blood lipid concentrations. In men, drink A tends to give a less health promoting response in the blood lipids with regard to cholesterol and LDL than in women. As side effects, four persons in group A and one person in group B reported minor gut discomfort such as pain or irregular movement.

**Table 6:** Differences in daily intake before and after the study period.

	**Group A before** **n = 27**	**Group A** **after** **n = 27**	**Difference (%)**	**Group B before** **n = 19**	**Group B after** **n = 19**	**Difference (%)**
Total energy intake (KJ)	8578 ± 399	8735 ± 367	+ 1,83	8502 ± 387	8898 ± 431	+ 4,66
Including testdrink		9496 ± 367	+ 10,7		9659 ± 431	+13,6

Cholesterol intake (mg)	291 ± 19	274 ± 20	-5,91	302 ± 30	290 ± 34	-3,97
Including testdrink		307 ± 20	+ 5,5		323 ± 34	+7,0

Saturated fat intake (g)	33,3 ± 2,3	33,8 ± 2,5	+1,56	33,9 ± 2,8	35,3 ± 2,6	+ 4,25

Total fat intake (g)	80,8 ± 4,9	83,2 ± 5,6	+2,89	83,1 ± 5,9	83,8 ± 4,6	+0,9
Including testdrink		87,4 ± 5,6	+6,6		88 ± 4,6	+5,9

The table shows intake of total energy, cholesterol and saturated fat in all participants. Differences before and after intake in each group was statistically analyzed using students paired t-test. * p < 0,01. ** p = < 0,001. *** p = < 0,0001. The values are mean ± SEM and the values for each subject are mean of four days of food registration before and the last four days.

## Discussion

This study investigates whether a daily intake of a SL-enriched milk drink affects fasting blood lipids in 48 subjects (33 men and 15 women) over 4 weeks. The background to this study was that SM was found to inhibit cholesterol absorption in rats [6] and the ability of several dietary SLs to decrease plasma cholesterol and TG and liver accumulation of TG in apoE Leyden mice on a fat- and cholesterol rich diet [8]. Another important factor was that a new formulation, prepared by a dairy technology that does not involve organic solvent extraction and is simple and cost effective, had become available.

The major finding in our study was that no significant plasma lipid lowering effects of the SL rich formulation A was found since there was no decrease in atherogenic plasma lipids or lipoproteins after the four week treatment.

There were, however, trends towards increased concentrations of total plasma cholesterol, LDL, HDL, apo B and TG in group B, which were not seen in group A.

To explain this finding an important aspect is that participants in both groups increased their energy intake and to some extent the intake of saturated fat and cholesterol (Table 6). It is not specified by the participants whether the test drink replaced a regular food item such as another milk product or was added to their normal diet, but according to their food registrations we believe that they mainly added the test drink. One can therefore argue that formulation A may have counteracted an increase in total cholesterol, apoB and TG, related mainly to an increased energy intake which provides some support for a positive effect of the milk polar lipids. ApoB/apoA1 ratios developed, however similar in the two groups (Figure 4).

Comparing the responses for the two formulations in men and women revealed that formulation B consistently increased many of the blood lipids whilst formulation A did not. Only the concentration of TG remained unaffected between the two groups. In group B the increase of TG was accounted for by men and the increased LDL and apoB concentrations were to a larger part accounted for by women. Women in group B were few and happened to have lower average starting concentration of TG than group A. This could make the TG response less sensitive to increased energy intake, which might decrease any difference between group A and B after four weeks. Overall, the variations we observe for the different blood lipids in the placebo groups after four weeks resemble effects that you get from intake of milk lipids rich in saturated fatty acids. This pattern was not observed in group A where, all together, very little effect from the test drink was seen.

Several studies have shown that the level of the independent risk factor Lp(a), although primarily genetically determined, may also be influenced by external factors. In milk context it was earlier reported that casein, in contrast to soy protein significantly decreased Lp(a)[12]. We therefore also compared the response of Lp(a) to the two formulations and found no difference between participants in group A and B. In women Lp(a) decreased, however, in response to the A- and increased to B formulation which was opposite the results in men where the increase was seen in A. Although not conclusive the study indicate sex differences that needs to be further investigated. Future studies should also consider that other components in milk might influence Lp(a).

There are several possible reasons why no clear positive effect of the SL formulation was seen on blood lipids in this study. For one thing, the outcome may reflect that SLs do not affect absorption of endogenous cholesterol which is necessary to achieve an effect on plasma cholesterol in humans. Older studies in which plant sterols were fed in an unesterified form provided little support for a significant cholesterol lowering effect, whereas plant sterol margarines in which sterol esters are mixed into dietary lipids are more effective [13]. Another factor is the dose given. In rodents digestion of SM is extended and has a limited capacity and the physical interaction between SM and cholesterol may be important. In contrast, humans express the key digestive enzyme for SM, alkaline sphingomyelinase also in the liver and secrets it in bile [14]. Sphingomyelin digestion might consequently be more efficient in humans.

In a recent study on ileostomy patients we found that after feeding 50-250 mg milk SM in a breakfast meal the overwhelming part had been digested and the cholesterol/SM ratios in the ileostomy content was high, although some increases in ileostomy output of SM species and ceramide were observed (L Ohlsson et al. submitted). The chosen dose of SL in this study (700 mg SM, 975 mg total, approx 1% of dietary lipids) is 2-3 times a normal Western dietary daily intake, which we believed large enough to test for possible effects. Considering the dose of plant sterols in margarines that is necessary to achieve decreases of total and LDL cholesterol, one may argue that the dose used here is not very high. Another factor is that SLs contain saturated fatty acids and the sphingoid bases are converted mainly to palmitic acid after absorption. They are thus a source of saturated fatty acids that raises LDL cholesterol although the quantity should not be sufficient to have significant influence in this study. Furthermore, both formulations contained a daily dose of 36 mg cholesterol which could be calculated to increase plasma cholesterol by 0,02-0,03 mmol/l [15].

Studies on apo BE receptor gene knockout mice, a model that is very sensitive to dietary cholesterol, suggested that SM-rich diet might raise plasma cholesterol levels and thereby enhance arteriosclerosis [16]. The finding in this study that LDL cholesterol exhibited some increase in the B but not in the A group indicate that these findings may not be relevant to healthy humans. Another important conclusion was that the formulations were both well tolerated and the only type of side effects reported were "Mild abdominal symptoms", which were reported by four persons in group A and one in B. We cannot exclude that the composition of formulation A may have contributed to this symptom, but the relation is uncertain since non specific symptoms like this can be caused by lactose even in individuals with normal lactase levels.

There is rather strong evidence that consumption of milk counteract colon cancer [17]. This effect may in part be ascribed to the calcium and vitamin D content [18]. Since several animal studies have found antitumor effects in the gut with SM or combinations of SL that occur in milk [19-21], there is reason to explore butter milk based formulations high in both calcium, vitamin D and polar lipids for anticarcinogenic and anti-inflammatory effects in human gut.

Polar milk lipids also contain phosphatidylcholine, phosphatidyl serine and smaller amounts of other glycerolipids. Studies with high doses of soy phospholipids have shown an LDL lowering effect, which may be due to high content of linoleic acid [22]. A minor raise in HDL_2 _by a high dose (15 g/day) of soy phospholipids was observed [23]. Consequently we do not expect the milk glycerophospholipids to exert any significant effect on the results in this study. However, a study by Sjogren et al [24]showed that milk consumption is associated with a more favourable size of the LDL particles. Further studies of SL enriched milk polar lipids on HDL and LDL composition may thus be motivated.

## Conclusion

This study did not provide strong proof of a lipid lowering effects of SL-enriched milk polar lipids, although it supports that formulation A counteracted TG and TDL increases related to an increased energy and fat intake. The SL rich formulation was well tolerated which promotes further studies that consider dose as well as synergies with other factors that may lower cholesterol absorption. Such studies should include hyper lipidemic patients and should consider also possible anti-inflammatory and anti-tumour effects in humans.

## Competing interests

Hans Burling is employed by Arla Foods who is the proprietor of patents related to the sphingolipid enriched butter milk formulation used in this study and who also provided partial economic support of the study. Authors LO, ÅN and HB have no other competing interests.

## Authors' contributions

LO carried out the practical work with the human study, analysis of results and wrote equal part of the manuscript as ÅN. HB provided the test drinks A and B, provided the main economical support for the study and drafted selected parts of the manuscript. ÅN participated in the design of the study, interpretation of the results and equal work as LO with the manuscript. All authors read and approved the final manuscript.
